# Editorial: Diagnostic, prognostic, and predictive markers in leukemia

**DOI:** 10.3389/fphar.2026.1908732

**Published:** 2026-09-03

**Authors:** Priyanka Sharma, Sitaramaraju Adduri, Sankaranarayanan Kannan, Hiroki Akiyama, Natthakan Thongon, Anudishi Tyagi, Weshely Kujur

**Affiliations:** 1 Department of Leukemia, The University of Texas MD Anderson Cancer Center, Houston, TX, United States; 2 Section of Molecular Hematology and Therapy, Department of Leukemia, The University of Texas MD Anderson Cancer Center, Houston, TX, United States; 3 Department of Cell and Molecular biology, University of Texas at Tyler Health Science Center, Tyler, TX, United States; 4 Department of Hematology, Institute of Science Tokyo, Tokyo, Japan; 5 Department of Internal Medicine, Massey Comprehensive Cancer Center, Virginia Commonwealth University, Richmond, VA, United States; 6 St. Jude Children’s Research Hospital, Memphis, TN, United States

**Keywords:** acute myeloid leukemia, biomarker, genetic, leukemia stem cells, metabolic, mitochondrial, molecular, tumor microenvironment

**FIGURE 1:**
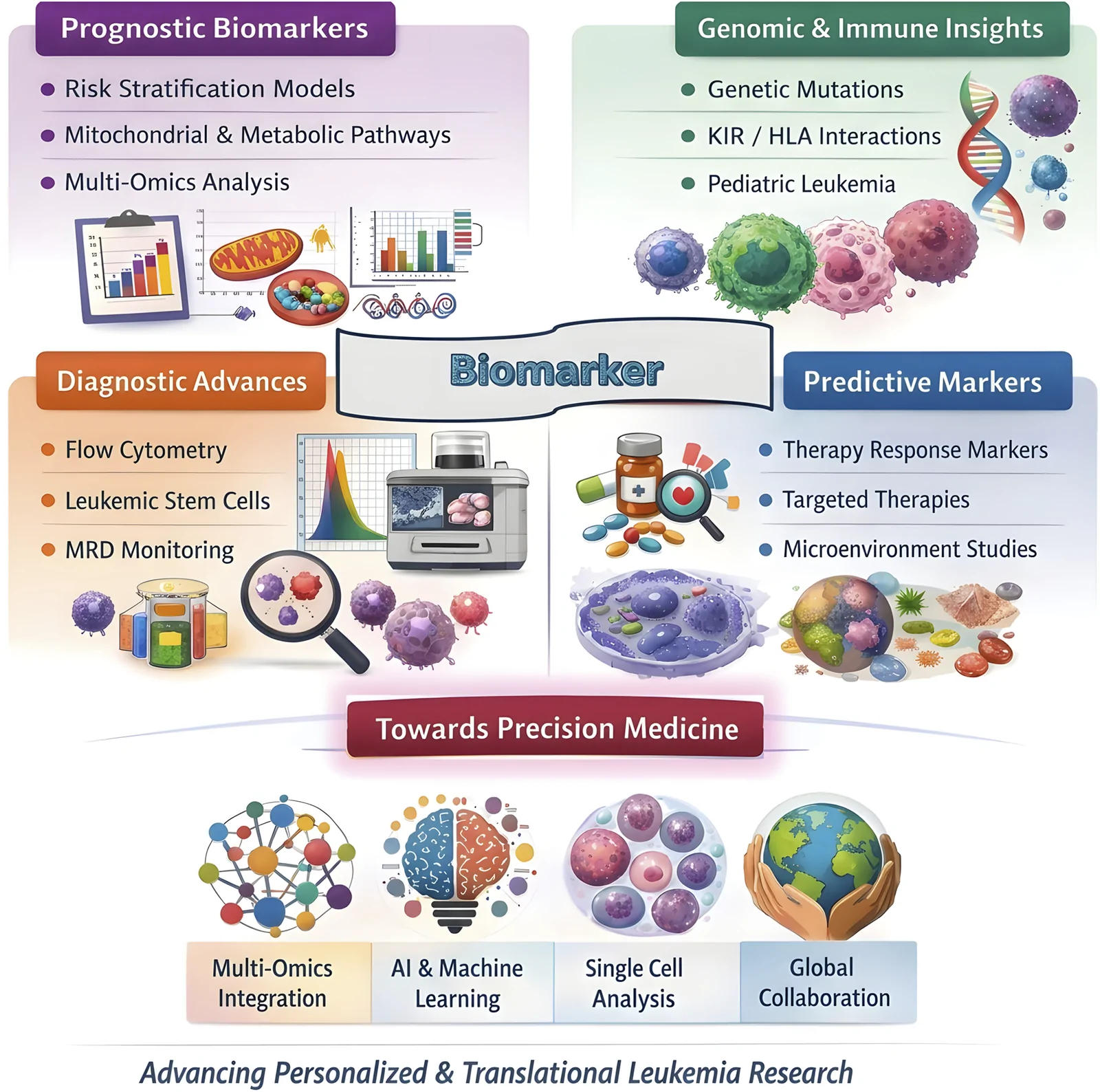
Integrative biomarker landscape in leukemia precision medicine. The schematic summarizes major biomarker domains highlighted in this Research Topic, including 1) prognostic biomarkers for risk stratification, mitochondrial and metabolic pathways, and multi-omics analysis; 2) genomic and immune insights involving genetic mutations and KIR/HLA interactions; 3) diagnostic advances such as flow cytometry, leukemic stem cell detection, and minimal residual disease monitoring; and 4) predictive markers related to therapy response, targeted therapies, and microenvironmental studies. Together, these approaches illustrate how multi-omics integration, artificial intelligence and machine learning, single-cell analysis, and global collaboration can advance translational leukemia research to guide personalized treatment.

Leukemia encompasses a diverse group of hematologic malignancies driven by complex interactions among genetic, epigenetic, metabolic, and microenvironmental factors. Although substantial advances in therapeutic strategies have improved outcomes for many patients, significant variability in disease progression and treatment response persists. This heterogeneity highlights the critical need for robust biomarkers that can refine diagnosis, improve prognostic accuracy, and guide therapeutic decision-making. The present Research Topic brings together 16 articles that collectively advance our understanding of diagnostic, prognostic, and predictive markers in leukemia, while also providing broader insights into disease biology and emerging therapeutic opportunities (Figure 1).

A major focus of this Research Topic lies in the development of prognostic biomarkers and risk stratification models, reflecting a shift toward integrative and systems-level approaches, combining clinical variables with molecular, metabolic, and proteomic data. Prognostic models based on mitochondrial function (Chen et al.), ribosome biogenesis (Chen et al.), and cell death pathways exemplify how non-traditional biological processes can inform patient outcomes. Chen et al. developed an interpretable ribosome biogenesis–based prognostic signature in acute myeloid leukemia (AML) using single-cell and machine learning approaches, demonstrating its ability to stratify patient outcomes and predict immune microenvironment features and immunotherapy response, while a separate study by Chen et al. established a machine learning–based mitochondrial risk score (MitoScore) integrating transcriptomic and single-cell data to refine prognosis, immune characterization, and drug sensitivity prediction in AML. Fan et al. and Zhu et al., based on 132 and 302 pediatric acute lymphoblastic leukemia (ALL) cases respectively, collectively demonstrated that fusion gene abnormalities are important determinants of disease biology, being strongly associated with immunophenotypic patterns, chromosomal alterations, minimal residual disease (MRD) dynamics, and clinical outcomes. Across both studies, *ETV6::RUNX1* and *E2A::PBX1* were consistently linked to favorable treatment response and improved survival, whereas *BCR::ABL* and *MLL* rearrangements were associated with high-risk disease features and inferior prognosis, underscoring the critical role of molecular fusion profiling in risk stratification, prognostic assessment, and individualized therapeutic decision-making in childhood ALL. In parallel, Cui et al. showed that clinically oriented scoring systems, such as the modified composite indices Endothelial Activation and Stress Index (EASIX), continue to demonstrate utility in predicting survival, particularly when adapted to pediatric leukemia. Notably, multi-omics approaches, including plasma proteomics (Hu et al.), further refine prognostic frameworks by simultaneously identifying biomarkers associated with survival and potential therapeutic targets. Together, these studies emphasize that contemporary prognostic modeling is moving beyond single-parameter assessments toward multidimensional platforms capable of capturing the complexity of leukemia biology.

Complementing these efforts are studies focused on molecular and genomic characterization, which form the foundation for both diagnostic and prognostic advancements. Investigations by Ruiz et al. into the genetic landscape of pediatric ALL across diverse populations reveal substantial heterogeneity in mutational profiles, emphasizing the need to incorporate region-specific genomic data to ensure accurate interpretation of biomarker signatures and to support their robust application across different demographic and geographic settings. Gao et al. further highlight the clinical relevance of disease heterogeneity by reporting a rare case of acute promyelocytic leukemia (APL) relapse occurring 18 years after complete remission, successfully treated with arsenic trioxide and all-trans retinoic acid, and emphasizing that very late relapse can still occur despite long-term remission, underscoring the need for continued vigilance and long-term follow-up in APL. These studies highlight the biological heterogeneity of leukemia, reinforcing the concept that it is not a single disease entity but a group of distinct subtypes that require tailored approaches.

In addition, Deng et al. analyzed immune-related genetic factors, including interactions between killer immunoglobulin-like receptors and human leukocyte antigen (KIR/HLA) systems, providing important insights into host–tumor interplay and potential protective mechanisms. These studies reinforce the notion that leukemia classification and risk assessment must account for both tumor-intrinsic and host-related factors.

Advances in diagnostic biomarkers and detection strategies are also represented within this Research Topic. Youssef and Park summarized the role of flow cytometric detection of AML leukemia stem cells (LSC), highlighting how high-resolution techniques including multiparametric flow cytometry are increasingly enabling the identification of rare but clinically significant cell populations such as leukemic stem cells in AML. The ability to detect and characterize these cells has important implications not only for diagnosis but also for understanding disease persistence and therapeutic resistance. Such approaches are likely to play an increasingly important role in refining disease classification and guiding treatment strategies.

The identification of predictive biomarkers, those capable of informing therapeutic response, remains essential for the advancement of precision medicine in leukemia. Contributions within this Research Topic explore signaling pathways and molecular markers that influence treatment sensitivity, particularly in chronic myeloid leukemia (CML) (Alqahtani), as well as multi-omics-derived biomarkers in AML that may serve as both predictors of response and targets for intervention. Gao et al. showed that decitabine inhibited T-ALL cell proliferation and induced apoptosis, associated with downregulation of PI3K, AKT, mTOR, 4EBP1 and upregulation of PTEN, highlighting modulation of the PI3K/AKT/mTOR pathway as a key biomarker-associated mechanism of response. Liu et al. showed that nilotinib plasma concentrations correlate with major molecular response in chronic myeloid leukemia, supporting therapeutic drug monitoring–guided dose optimization with defined efficacy and toxicity thresholds for personalized treatment. These studies highlight the growing convergence between biomarker discovery and therapeutic development, where molecular insights are increasingly informing strategies for patient stratification and targeted treatment.

An emerging and particularly important theme is the role of the tumor microenvironment and systems biology in shaping disease behavior. Several studies adopt integrative approaches that incorporate immune profiling and circulating biomarkers, thereby expanding the scope of biomarker research beyond tumor-intrinsic features to include host and microenvironmental factors. Notably, Chen et al. linked their ribosome biogenesis–based risk score to distinct immune microenvironment states, with high-risk patients showing an immunosuppressive milieu enriched for Tregs and M2 macrophages, reduced cytotoxic T/NK cells, and differential cytokine/chemokine patterns characterized by increased CCL16, CX3CL1, VEGFB, and CXCL14 alongside decreased IFN-γ–associated signaling. These immune features further correlated with predicted immunotherapy response, underscoring the relevance of integrating immune and inflammatory mediators into prognostic models. Tsagiopoulou et al. show that activation of MYC target genes is linked to aggressive disease biology in chronic lymphocytic leukemia, particularly in unmutated IGHV cases, trisomy 12, and Richter transformation. This activation correlates with increased proliferation, enhanced microenvironmental interactions, and shorter time to first treatment, highlighting *MYC* as a potential biomarker for risk stratification and a candidate therapeutic target. As such, microenvironmental and systemic factors are increasingly recognized as critical components of comprehensive biomarker models.

Finally, a subset of articles provides mechanistic and conceptual insights into leukemia biology. Reviews and experimental studies focusing on signaling pathways, epigenetic regulators such as *KMT2D* (Schlemminger et al.), and biologically complex leukemias such as mixed phenotype acute leukemia (Karasek et al.) offer a broader framework for interpreting biomarker findings within the context of underlying disease mechanisms. Such integrative perspectives are essential for bridging the gap between descriptive biomarker studies and actionable biological understanding, ultimately facilitating the translation of research findings into clinical applications.

Collectively, the articles in this Research Topic reflect a broader paradigm shift in leukemia research. There is an increasing emphasis on integrative strategies that leverage multi-omics data, computational modeling, and systems biology rather than relying on individual biomarkers. This evolution has the potential to enable more precise disease classification, improved risk stratification, and the identification of novel therapeutic targets. At the same time, these advances highlight several ongoing challenges, including the need for robust validation in large, independent cohorts, the standardization of analytical methodologies, and the effective integration of biomarker data into routine clinical practice.

Looking ahead, future research should prioritize the clinical translation and implementation of biomarker discoveries. Prospective clinical trials incorporating biomarker-driven stratification will be essential for demonstrating clinical utility and determining whether these approaches can improve patient outcomes. The integration of artificial intelligence and machine learning approaches offers significant potential for handling complex, high-dimensional datasets and may improve predictive modeling with enhanced accuracy. In addition, emerging technologies such as single-cell sequencing and spatial transcriptomics are likely to further refine our understanding of leukemia heterogeneity and microenvironmental interactions.

Another critical area for future investigation is the advancement of minimal residual disease (MRD) monitoring, which remains among the most powerful predictors of relapse. Although MRD assessment by multiparametric flow cytometry remains clinically relevant, its sensitivity is limited by immunophenotypic overlap between leukemic stem cells and normal hematopoietic stem cells, as well as the heterogeneity of leukemic populations. Therapy-resistant subclones are genetically and functionally distinct leukemia cell populations enriched for stem cell–like features, quiescence, stress-adaptive signaling, and anti-apoptotic programs that enable survival during chemotherapy. These residual populations are often undetectable by conventional MRD assays yet retain the capacity to drive relapse. To address these challenges, Youssef and Park propose integrating refined LSC detection approaches to improve MRD accuracy by capturing resistant compartments. Collectively, this supports the evolution of MRD frameworks toward more sensitive, stem cell–informed strategies for improved prognostic precision in AML. Longitudinal studies using single-cell technologies, that track biomarker dynamics over the course of disease and treatment will be essential for understanding clonal evolution and mechanisms of resistance.

Importantly, ensuring equitable representation of diverse patient populations in genomic and biomarker studies will be critical for the global applicability of these advances. Differences in genetic background, environmental exposures, and healthcare access can all influence biomarker performance and clinical outcomes. As such, global collaboration and data sharing will likely play a key role in advancing the field.

In conclusion, this Research Topic highlights a continuing shift toward more precise and personalized leukemia care through the integration of molecular, clinical, and systems-level insights. Continued validation and clinical translation of these findings will be essential to improve patient outcomes. In addition, the timing and frequency of sample collection, together with the temporal resolution of biomarker analyses, represent important but often underrecognized factors that can influence the accuracy of diagnostic and prognostic assessments.

